# The Viruses of Wild Pigeon Droppings

**DOI:** 10.1371/journal.pone.0072787

**Published:** 2013-09-04

**Authors:** Tung Gia Phan, Nguyen Phung Vo, Ákos Boros, Péter Pankovics, Gábor Reuter, Olive T. W. Li, Chunling Wang, Xutao Deng, Leo L. M. Poon, Eric Delwart

**Affiliations:** 1 Blood Systems Research Institute, San Francisco, California, United States of America; 2 Department of Laboratory Medicine, University of California San Francisco, San Francisco, California, United States of America; 3 Pharmacology Department, School of Pharmacy, Ho Chi Minh City University of Medicine and Pharmacy, Ho Chi Minh, Vietnam; 4 Regional Laboratory of Virology, National Reference Laboratory of Gastroenteric Viruses, ÁNTSZ Regional Institute of State Public Health Service, Pécs, Hungary; 5 Stanford Genome Technology Center, Stanford, California, United States of America; 6 Centre of Influenza Research and School of Public Health, University of Hong Kong, Hong Kong SAR; Duke-NUS Gradute Medical School, Singapore

## Abstract

Birds are frequent sources of emerging human infectious diseases. Viral particles were enriched from the feces of 51 wild urban pigeons (*Columba livia*) from Hong Kong and Hungary, their nucleic acids randomly amplified and then sequenced. We identified sequences from known and novel species from the viral families *Circoviridae, Parvoviridae, Picornaviridae, Reoviridae, Adenovirus, Astroviridae,* and *Caliciviridae (listed in decreasing number of reads)*, as well as plant and insect viruses likely originating from consumed food. The near full genome of a new species of a proposed parvovirus genus provisionally called *Aviparvovirus* contained an unusually long middle ORF showing weak similarity to an ORF of unknown function from a fowl adenovirus. Picornaviruses found in both Asia and Europe that are distantly related to the turkey megrivirus and contained a highly divergent 2A1 region were named mesiviruses. All eleven segments of a novel rotavirus subgroup related to a chicken rotavirus in group G were sequenced and phylogenetically analyzed. This study provides an initial assessment of the enteric virome in the droppings of pigeons, a feral urban species with frequent human contact.

## Introduction

Many infectious diseases in humans are caused by pathogens originating from a wide variety of animals. More than 60% of emerging diseases are estimated to originate from wildlife [1]–[4]. Public awareness of zoonoses has recently increased because of their public health and economic impacts. Birds are recognized as frequent reservoirs for viruses that are of concern to humans; notably influenza A which is capable of infecting other mammals thereby facilitating genome segment reassortments and changes in tropism and transmission efficiency [5]–[8]. Sporadic human infections of the virulent H5N1 resulting from direct contact with infected poultry or wild birds have been reported in 15 countries, mainly in Asia [9]–[13], and H7N9 has recently emerged as a virus of concern. The prevalence of avian influenza viruses was 12% of oropharyngeal and 20% of cloacal swab specimens collected from urban pigeons in Slovakia [14]. H5N1 was found in a dead feral pigeon in Hong Kong [15] but is generally apathogenic in this host species and the overall risk of H5N1 transmission from pigeons to humans or chickens appears low [16], [17]. West Nile virus (WNV) and Saint Louis encephalitis (SLE) virus, two arboviruses in the *Flavivirus* genus transmitted by mosquitoes bites, are disseminated by wild birds [18]–[23]. WNV-specific antibody and viremia was found in 25.7% and 11% of rock pigeons, respectively in the United States [24]. WNV was also isolated in pools of brains, kidneys, heart and spleen of feral pigeons and mapgies [25]. Pigeons developed low levels of WNV viremia; insufficient to infect mosquitoes [24], [26]. Avian paramyxoviruses, including Newcastle disease virus, are common domestic and wild bird pathogens [27]–[30]. Paramyxovirus type-1 can be found in pigeons worldwide [31]–[34] but the clinical signs vary depending on the immunity of the host and virulence of the specific isolates [35]. While human infection with Newcastle disease virus is rare, at least two outbreaks of conjunctivitis due to Newcastle disease virus have been reported in poultry workers [28], [36], [37]. Chicken anemia virus (CAV), until recently the only member of the gyrovirus genus, is highly contagious and causes severe anemia, hemorrhage and depletion of lymphoid tissue in chickens [38]–[40]. Related gyroviruses were recently characterized in human feces, blood and on healthy human skin [41]–[44] indicating possible human tropism. Gyrovirus DNA was also detected in three blood samples of solid organ transplant patients and in one HIV-infected person [44] as well as in 0.85% of healthy French blood donations [45].

Pigeons are therefore natural reservoirs for pathogens that have caused emerging and re-emerging diseases in humans. In order to better understand the viruses shed by pigeons to which humans are frequently exposed, we genetically characterized the viral community in droppings from wild pigeons in Hong Kong and Hungary following an unbiased amplification method and deep sequencing.

## Materials and Methods

### Biological Samples and viral Metagenomics

51 fecal specimens were collected from feral pigeons (*Columba livia*) in Hong Kong (N = 50) in August 2011 and in Pécs, Hungary (N = 1) in April 2011, then stored at −80°C. Droppings were collected without any physical contact with wild urban pigeons. Procedure therefore did not require animal committee ethical review. The field studies did not involve endangered or protected species. Fresh and well-separated droppings were sampled using sterile swabs which were stored in two ml of viral transport media as described [46]. The fecal sample from Pécs, Hungary was collected from a nesting 5–8 days old, clinically healthy pigeon into Hanks’ buffered saline solution (Gibco BRL). Prior to the shipment on dry ice, samples were heat treated at 74°C for 50 minutes to inactivate viruses. The suspensions were vigorously vortexed and clarified by 15,000 × *g* centrifugation for 10 minutes. 200 µl of the supernatant was filtered through a 0.45-µm filter (Millipore) to remove bacterium-sized particles. The filtrate was then treated with a mixture of DNases (Turbo DNase from Ambion, Baseline-ZERO from Epicentre, and Benzonase from Novagen) and RNase (Fermentas) to digest unprotected nucleic acids [47]. Viral nucleic acids protected from digestion within viral capsids and other small particles were then extracted using a QIAamp spin-column technique according to the manufacturer’s instructions (Qiagen). For the single fecal specimen from Hungary, non-specific RNA and DNA amplification was then performed by random RT-PCR using a primer with randomized 3′ ends [47]. Amplicons were then pooled, 454 libraries generated according to the manufacturer’s protocol (GS FLX Titanium General Library Preparation Kit, Roche) and pyrosequenced using the 454 Titanium FLX+ sequencer. For the 50 fecal specimens from Hong Kong, pools of nucleic acids from five fecal specimens were generated resulting in ten separate pools. A library was then constructed using ScriptSeq™ v2 RNA-Seq Library Preparation Kit (Epicentre) and then sequenced using the Miseq Illumina platform with 250 bases paired ends with a distinct molecular tag for each pool. Because fecal specimens were analyzed in pools of five, the viruses identified are reported per specimen pool rather than for individual specimens. The 454 and Illumina singleton reads and assembled contigs greater than 100-bp were compared to the GenBank protein databases using BLASTx. An E value of 10^−5^ was used as the cutoff value for significant hits. The sequence data from the MiSeq run is in the short read archive at GenBank accession number SRX263026.

### Genome Acquisition of Novel Viruses

Sequences showing significant but divergent BLASTx hits to parvoviruses, picornaviruses or rotaviruses in the same sample pool were linked together using RT-PCR or PCR and primers based on the initial short sequence reads or contigs. 5′ and 3′ rapid amplification of cDNA end (5′ and 3′ RACE) was used to acquire the 5′ and 3′ extremities of viral genome. Phylogenetic analyses were performed using novel virus sequences, their best BLASTx hits, and representative members of related viral species or genera. All alignments and phylogenetic analyses were based on the translated amino acid sequences. Sequence alignment was performed using CLUSTAL X (version 2.0.3) with the default settings [48]. Aligned sequences were trimmed to match the genomic regions of the viral sequences obtained in the study. A phylogenetic tree with 100 bootstrap resamples of the alignment data sets was generated using the neighbor-joining method based on the Jones-Taylor-Thornton matrix-based model in MEGA version 5 [49]. Bootstrap values (based on 100 replicates) for each node are given if >70%. The generated phylogenetic trees were visualized using the program MEGA version 5. Resulting trees were examined for consistency with published phylogenetic trees. Sequence identity was measured using BioEdit [50]. The sequence distance comparison was calculated by using SSE [51]. Conserved amino acid analyses were performed over the alignments produced by CLUSTAL X (version 2.0.3). The hypothetical cleavage map of the picornavirus polyprotein was derived from alignments with other closest picornaviruses and NetPicoRNA prediction. Putative ORFs in the genome were predicted by NCBI ORF finder. The secondary structure of 5′ and 3′ UTR was predicted using the Mfold program.

### Bioinformatics Analysis

We received 36.5 million 250-bp paired-end reads generated on the Illumina Mi-Seq platform. Short reads were debarcoded using vendor software from Illumina. A total of 6,818 reads from 454 pyrosequencing were trimmed of their primer sequence and adjacent eight nucleotides corresponding to the randomized part of the primer. In-house analysis pipelines were developed to process both datasets. Adaptors were trimmed by in-house code. The cleaned reads were de-novo assembled using SOAPdenovo2 (38). The assembled contigs, along with singlets, were aligned to an in-house viral protein database using NCBI BLASTx. The significant hits to viral sequences were then aligned to an in-house non-virus-non-redundant (NVNR) universal protein database using BLASTx. Hits with more significant adjusted E-value to NVNR than to virus were removed.

## Results

### Viral Metagenomic Overview

The distributions of the viral hits among the ten pools of five fecal samples from Hong Kong are shown in Table 1 based on BLASTx E scores <10^−5^. A range of best hits (BLASTx E scores <10^−5^) to different viral families infecting plants (*Germinividae, Nanoviridae, Caulimoviridae, Hypoviridae, Umbravirus, Mycovirus, Totiviridae, Tombusviridae*) and insects (*Iflavirus, Tetnovirus, Chronic bee paralysis virus, Laem Singh virus*) were detected (Table 1). The mammalian viral hits belonged to the *Circoviridae*, *Parvovirinae* subfamily, *Picornavirus*, *Rotavirus*, *Adenovirus*, *Astrovirus*, and *Calicivirus*. The *Circoviridae*-related sequences showed best hits to three different clades, *Circovirus* (1824 reads), *Cyclovirus* (20 reads) and *Gyrovirus* (2 reads). 77% of the circovirus hits showed best matches to pigeon circovirus of which 108 sequences had >90% aa-identity to pigeon circovirus. 74% of the *Parvovirinae* sequences showed best hits to AAV (*Adeno-associated virus*). A majority of the picornavirus sequences (85%; 759/889) showed best hits to *Turkey hepatitis virus* [52]. The adenovirus sequences showed best hits to three difference genera *Mastadenovirus* (3 reads), *Aviadenovirus* (77 reads) and *Siadenovirus* (296 reads).

**Table 1:** Distribution of sequence reads to different viral species/families in 10 sample pools from Hong Kong and the single sample from Hungary.

No. of reads	Sample ID	Mammalian							Insect				Plant									
		Circoviridae	Parvoviridae	Picornavirus	Rotavirus	Adenovivus	Astrovirus	Calicivirus	Tetnovirus	CBPV	LSV	Iflavivirus	Geminiviridae	Tobamovirus	Nanoviridae	Tombusviridae	SsHADV-1	Hypovirus	Totiviridae	Caulimoviridae	SmV A	Umbravirus
**22438**	**1**	75	41	29	2	31							7	3			2		3			
**39193**	**2** *	378	**105**	63									4		1							
**23643**	**3**	81	89	162		14					2	1	1					6				
**19787**	**4** *	98	307	133	**461**	4				3							4					
**90660**	**5** *	121	203	**205**	2	14	2				1		8	5	8	11				1		
**8794**	**6**	425	281	53		4		5	33					1		7						1
**60389**	**7**	100	41	50		6	35						2		2		3			1		
**42138**	**8**	153	129	24	3	75								16	2							
**51485**	**9**	154	198	133		8	4						15	2	2						2	
**75010**	**10**	261	291	20	117	220	1						16	5	9		1					
**6818**	**GALII.5** *			**17**			8							1								
**440355**	**Total**	1846	1685	889	585	376	50	5	33	3	3	1	53	33	24	18	10	6	3	2	2	1

* Sample contained the initial novel parvovirus, rotavirus or picornavirus sequence reads that were further analyzed in the study.

SmV A: Sclerophthora macrospora virus A; CBPV: Chronic bee paralysis virus; SsHADV-1: Sclerotinia sclerotiorum hypovirulence-associated DNA virus 1; LSV: Laem Singh virus.

The pigeon fecal sample from Hungary was analyzed using 6,818 unique reads from 454 pyrosequencing and showed viral hits to picornaviruses, astroviruses, and tobamoviruses.

We further characterized a subset of divergent mammalian viruses by acquiring their nearly complete or full genomes and phylogenetically comparing them to their closest viral relatives.

### Proposed New Avian Parvovirus Genus

Parvoviruses are small non-enveloped viruses with linear single-stranded DNA genomes of approximately 5-kb. The family *Parvoviridae* is classified into two subfamilies, *Parvovirinae* and *Densovirinae*, which infect vertebrates and invertebrates, respectively [53]–[57]. The subfamily *Parvovirinae* currently consists of five genera *Bocavirus*, *Erythrovirus*, *Dependovirus*, *Amdovirus* and *Parvovirus*; however, recently discovered genomes are expected to greatly increase that number [57], [58]. Parvoviruses are widespread pathogens that can cause a range of diseases in a variety of mammals as well as birds and reptiles [57].

**Figure 1:**
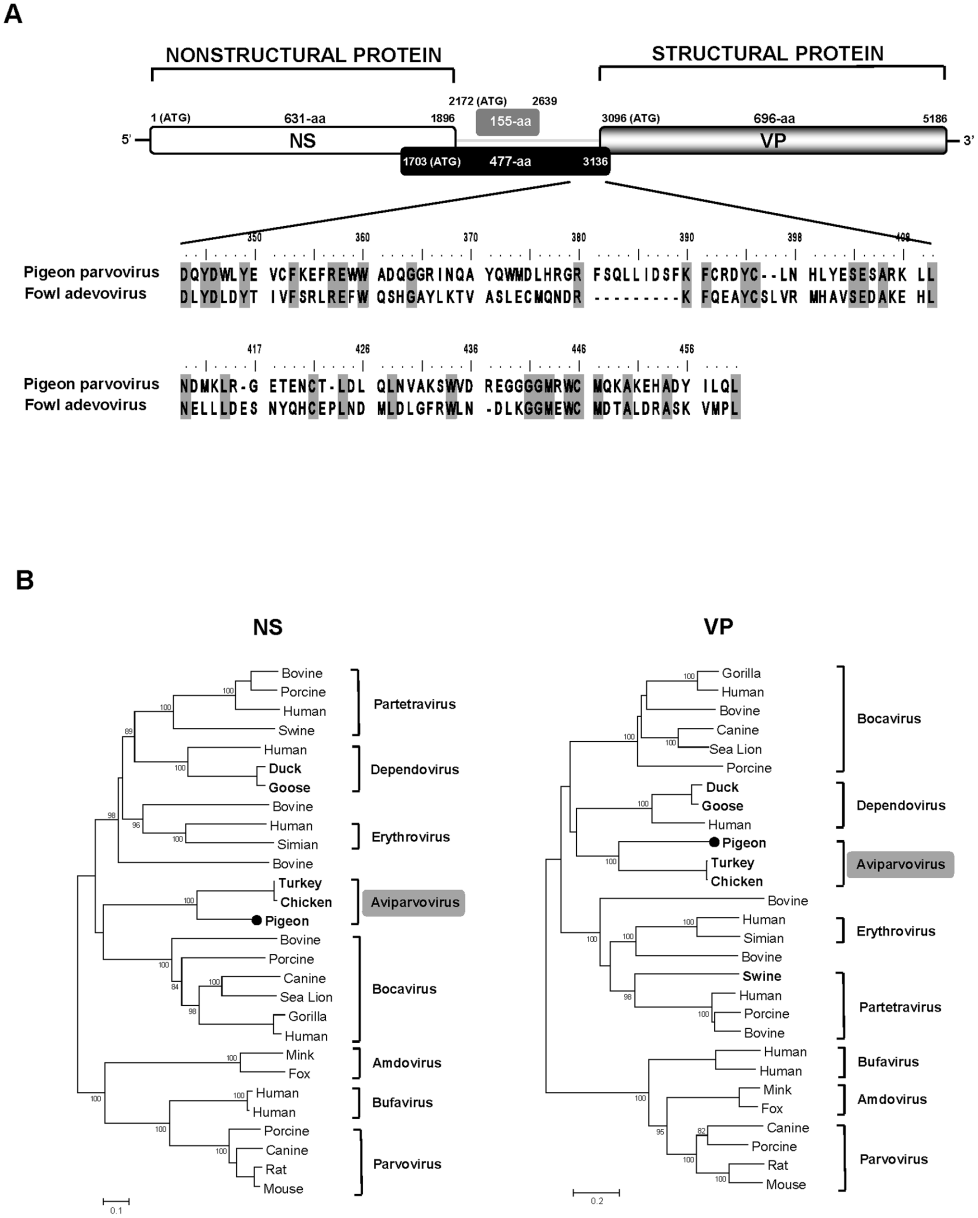
Pigeon parvovirus genome and phylogeny. **A.** Genome organization of pigeon parvovirus. The alignment of the large central ORF of pigeon parvovirus and ORF78 of fowl adenovirus is shown. **B.** Phylogenetic analyses of NS and VP proteins of pigeon parvovirus and related parvoviruses. The scale indicated amino acid substitutions per position. ICTV approved and proposed *Parvovirinae* genera are shown with those containing avian parvoviruses labeled in bold font. GenBank accession numbers used are included in Table S3.

One hundred and five out of a total of 39,193 reads from one specimen pool (Table 1 pair 2) were related to parvovirus proteins (BLASTx E scores: 7×10^−6^ to 5×10^−41^). Gaps between short sequence reads were filled by PCR and then directly Sanger-sequenced. The nearly complete genome (5492-bp) of pigeon parvovirus, including a partial 5′ UTR (252-bp), complete NS (631-aa), complete VP (696-aa) and a partial 3′ UTR (54-bp) was acquired (GenBank KC876004). As expected the genome contained two large open reading frames (ORFs) with the left and right ORFs encoding non-structural (NS) and viral capsid protein (VP), respectively (Figure 1A). Distinct from its closest genetic relatives (chicken and turkey parvoviruses), pigeon parvovirus A contained an unusually long middle ORF (477-aa) (Figure 1A). The carboxy termini of this ORF (135-aa; position 343–477) showed best although weak similarity (BLASTx to NR E score: 8.9×10^−2^) to a segment of a 221-aa ORF43 predicted encoded protein from the large dsDNA fowl adenovirus genome [59], sharing 28% amino acid identity (Figure 1A). A smaller middle ORF (155-aa) was also detected, which did not show similarity to any other sequence in GenBank. The start codon of NS was located in a strong Kozak sequence context, CAGCATGGC and contained the ATP or GTP binding Walker loop motif ^401^GPANTGKT^408^ [GXXXXGK(T/S)] [60]. Additionally, two conserved replication initiator motifs ^118^RC**H**V**H**IMLI^126^ and ^145^ITK**Y**VTEALT^154^ were also found [61]. Similar to chicken parvovirus, pigeon parvovirus’s VP protein did not possess the phospholipase A_2_ (PLA_2_) motif with its highly conserved calcium-binding site (YLGPF) as well as the phospholipase catalytic residues (HD and D). The N-terminus of its VP protein contained glycine rich sequence (GGGGSVGSGGGGGVG) also present in other parvoviral VP proteins. Pair-wise amino acid sequence analysis showed that the NS and VP proteins in pigeon parvovirus shared the highest aa-identities of 41% and 34% to those of chicken parvovirus [62], and less than 20% to the proteins of other parvovirus genera (Table S1). Figure 1B shows that pigeon parvovirus NS and VP proteins shared a monophyletic root with chicken and turkey parvoviruses, indicating a common origin. Based on the NS and VP phylogenetic analysis and genetic distance calculations, parvoviruses from pigeons, chickens and turkeys may be included as members of as a novel genus with a proposed name of *Aviparvovirus* (for avian parvovirus) in the subfamily *Parvovirinae*.

### Pigeon Picornavirus

Picornaviruses are small, non-enveloped, single-stranded RNA viruses whose prototype is poliovirus. The family *Picornaviridae* currently consists of seventeen genera (http://www.picornaviridae.com) that infect a wide range of hosts including mammalian and bird species. Many other recently characterized picornavirus genomes have increased the number of potential *Picornaviridae* genera [63]–[66]. The genome of a typical picornavirus ranges from 7 Kb to 9 Kb and, with one exception, contains a single long ORF coding for a polyprotein [67]. Picornaviruses have been reported in fecal and respiratory specimens from numerous vertebrates including humans, bats, rodents, pigs, fish and reptiles [65], [68]–[70].

**Figure 2:**
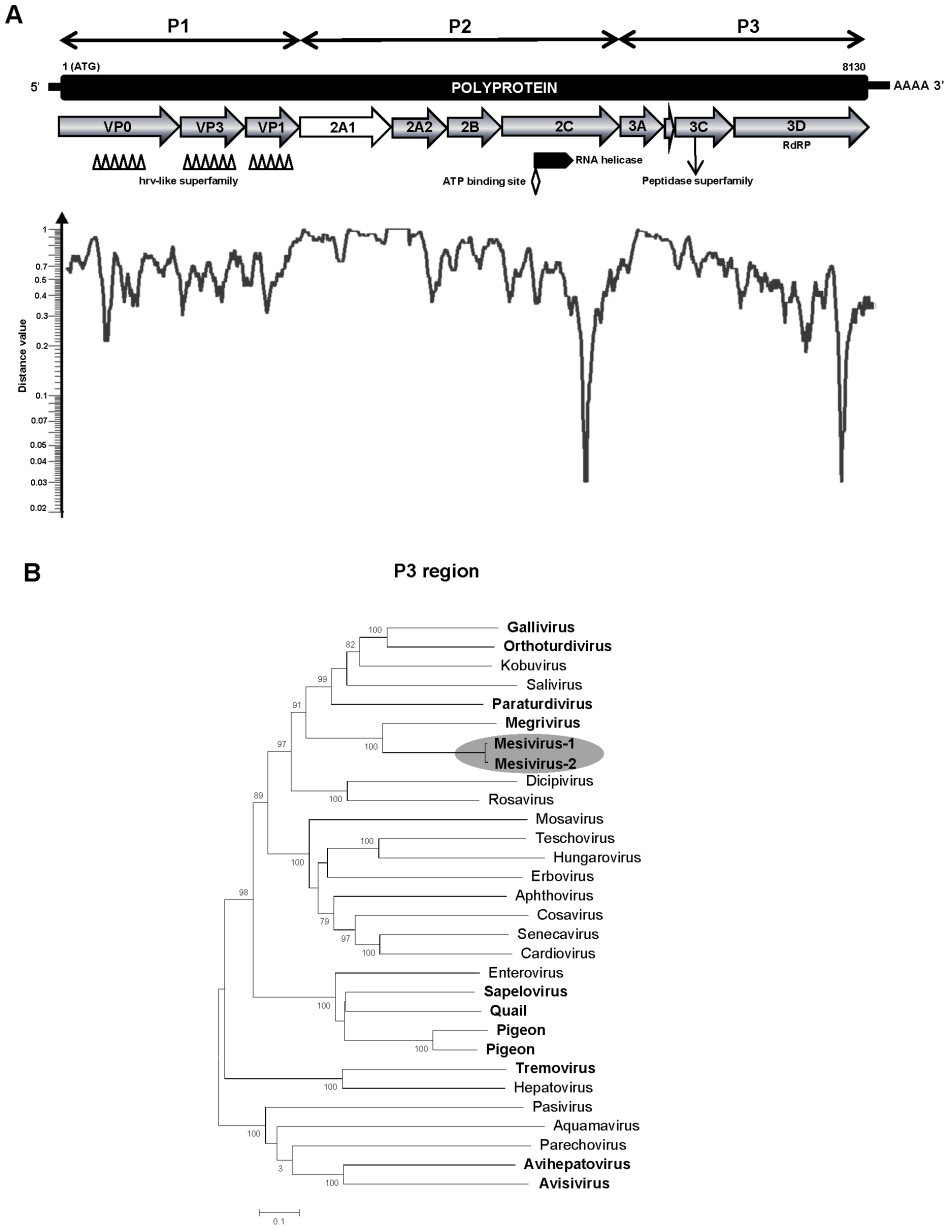
Mesivirus genome and phylogeny. **A.** Genome organization of *Mesivirus* (*Picornaviridae*) and its sequence distance to the closest genetic relative, the turkey hepatitis virus (HQ189775) belonging to genus *Megrivirus*. **B.** Phylogenetic analyses of P3 regions of *Mesivirus-1* (KC876003) from Hong Kong and *Mesivirus-2* (KC811837) from Hungary and other representative picornaviruses. ICTV approved and proposed *Picornaviridae* genera are shown with those containing avian picornaviruses labeled in bold font. GenBank accession numbers used are included in Table S4.

In this study we characterize two complete genomes of a novel picornavirus from pigeon fecal samples collected from Hong Kong (strain HK-21, KC876003) and Hungary (strain GALII-5/2011/HUN, KC811837) with a 9,072/9,192 nucleotide (nt) long genome [excluding the poly(A) tail] (Figure 2A). The P1, P2 and P3 regions of GALII-5/2011/HUN showed 74%, 92% and 97% amino acid identity to strain HK-21 indicating a presence of two genotypes of the same picornavirus species. Polyprotein alignments revealed that strain GALII-5/2011/HUN had an alternative upstream start codon **M**REY instead of the putative start codon of **M**ATF seen in strain HK-21 (Figure S2). A stop codon was found between **M**REY and **M**ATF in strain HK-21 but none was found in strain GALII-5/2011/HUN. The start codon in the upstream **M**REY in GALII-5/2011/HUN was also located in a weaker Kozak consensus sequence [GGGGATGCG] which plays a major role in the initiation of the translation process than **M**ATF [GGAGATGGC] (Figure S2). We therefore selected the second methionine codon (**M**ATF) as the start codon for the analysis. The single 2,707/2,711-aa-long (of HK-21/GALII-5/2011/HUN) polyprotein coding region was flanked by the 620/727 nt-long 5′ UTR and the 320/332-nt-long 3′ UTR. By BLASTx, its polyprotein showed the closest identity to the turkey hepatitis virus belonging to *Megrivirus* genus [52]. Therefore, we provisionally named these piconaviruses as *Mesivirus-1* (strain HK-21) and *Mesivirus-2* (strain GALII-5/2011/HUN) for Megrivirus sister-clade virus.

The mesivirus 5′ UTR contained a highly conserved, 20 nucleotides long motif and apical “8”-like structure ^407^GGAGG**TGGTGCTGAAATATTGCAAG**CCACT^437^ (with unknown function) that were also seen in avian origin picornaviruses, including turkey hepatitis virus (genus *Megrivirus*), duck hepatitis A virus-1 (genus *Avihepatovirus*), quail picornavirus (unassigned genus) and pigeon picornavirus B (unassigned genus) [52], [71], [72]. All these picornaviruses had type IV-like IRES [http://www.picornaviridae.com], however, we could not draw the potential secondary structure of mesivirus IRES. The hypothetical cleavage map of the polyproteins of mesiviruses were derived from alignments with other known picornaviruses and NetPicoRNA prediction [73]. Mesiviruses did not have a putative L protein preceding the capsid region. The P1 regions were 807/809-aa (*Mesivirus-1/Mesivirus-2*) in length containing rhv-like superfamily domains (Figure 2A) revealed by Conserved Domain Database (CDD) search [74]. The conserved motif GXXXT/S for myristylation was also absent. Similar to the megrivirus, there was no potential VP2/VP4 cleavage site in mesiviruses. The P1 polyproteins of mesivirus-1 and -2 could be theoretically cleaved at VP0/VP3 (Q^389–390^↓Y/T), VP3/VP1 (Q^557/558^↓G) and VP1/2A1 (Q^807/809^↓D/E). BLASTx showed that the P1 region had the highest aa-identity of 43%-41% to the megrivirus (strain 0091.1, HQ189775) (Table S2). The P2 polypeptides (1019/1021-aa) encoded non-structural proteins of mesivirus-1 and -2 cleaved at 2A1/2A2 (G^1095/1099^↓R), 2A2/2B (Q^1290/1294^↓A), 2B/2C (E^1480/1484^↓A) and 2C/3A (E^1826/1830^↓A) (Table S2). Similar to the megrivirus Turkey hepatitis virus, both mesivirus genomes encoded an unusual 2A1 (288/290 aa) at the N-terminal end of the P2 polypeptide. When megrivirus and mesivirus polyproteins where aligned their 2A1 regions showed the weakest homology along their genomes with only the central part of 2A1 (28%) showing 31% identity to that of the megrivirus 2A1 strain 0091.1 (accession number HQ189775) (Figure S1A). The 195-aa-long 2A2 proteins of both mesiviruses contained an HBox/NC domain (H^1127/1131^ WG and N^1188/1192^ CT) that was also observed in the 199-aa-long 2A2 of megriviruses [75]. Characteristic features of picornaviruses, including the RNA-helicase superfamily and NTPase motifs, were identified in the mesivirus 2C proteins. The P3 of megriviruses were 881-aa in length encoding proteins 3A, 3B (VPg, small genome-linked protein), 3C^pro^ (protease) and 3D^pol^ (RNA-dependent RNA polymerase). The P3 polypeptides were cleaved at 3A/3B (E^2008/2012^↓A), 3B/3C (E^2036/2040^↓G) and 3C/3D (Q^2233/2237^↓G) (Table S2). The 3C^pro^ contained the H-D-C catalytic triad and conserved GXCG [G^2190/2194 ^FCG] motif known as an active site of protease [76]. The 3D^pol^ contained conserved RdRp motifs K^2398/2402^ ELR, Y^2567/2571^ GDD and F^2616/2620^ LKR. It also had a conserved motif G^2527/2531^ GMPSG that was also seen in other picornaviruses [77].

Phylogenetic analysis of P1–P3 also showed that mesiviruses were most closely related to megrivirus (Figure 2B and Figure S1B) and in pair-wise comparison mesivirus-1/mesivirus-2 had identities of 43%/41%, 34%/30% and 47%/47% to turkey megrivirus (strain 0091.1) at the amino acid level for the P1, P2 and P3 regions respectively (Table S2). According to the ICTV, the member of a picornavirus genus should share >40%, >40% and >50% aa-identities in P1, P2 and P3 regions respectively (http://www.picornaviridae.com). Although the identities of the P2 and P3 regions qualified mesiviruses as belonging to a novel genus, its P1 region shared more than the 40% aa-identity threshold to megrivirus. Mesiviruses might therefore be considered as a new picornavirus species in the genus *Megrivirus* or given their distinct 2A1 and high P2 and P3 divergence to megrivirus as members of a separate genus.

### Pigeon Rotavirus

Rotaviruses consist of at least eight groups or species (A through H) with multiple P and G genotypes which together comprise a genus in the family *Reoviridae*. Rotavirus has a non-enveloped, triple-layered icosahedral capsid containing eleven segments of double-stranded RNA, encoding for six structural and five nonstructural proteins [78]–[80]. We successfully acquired 17,827 nucleotides of this highly divergent rotavirus (HK18), encoding the complete proteins of all 11 genome segments (GenBank KC876005–KC876015).

**Figure 3:**
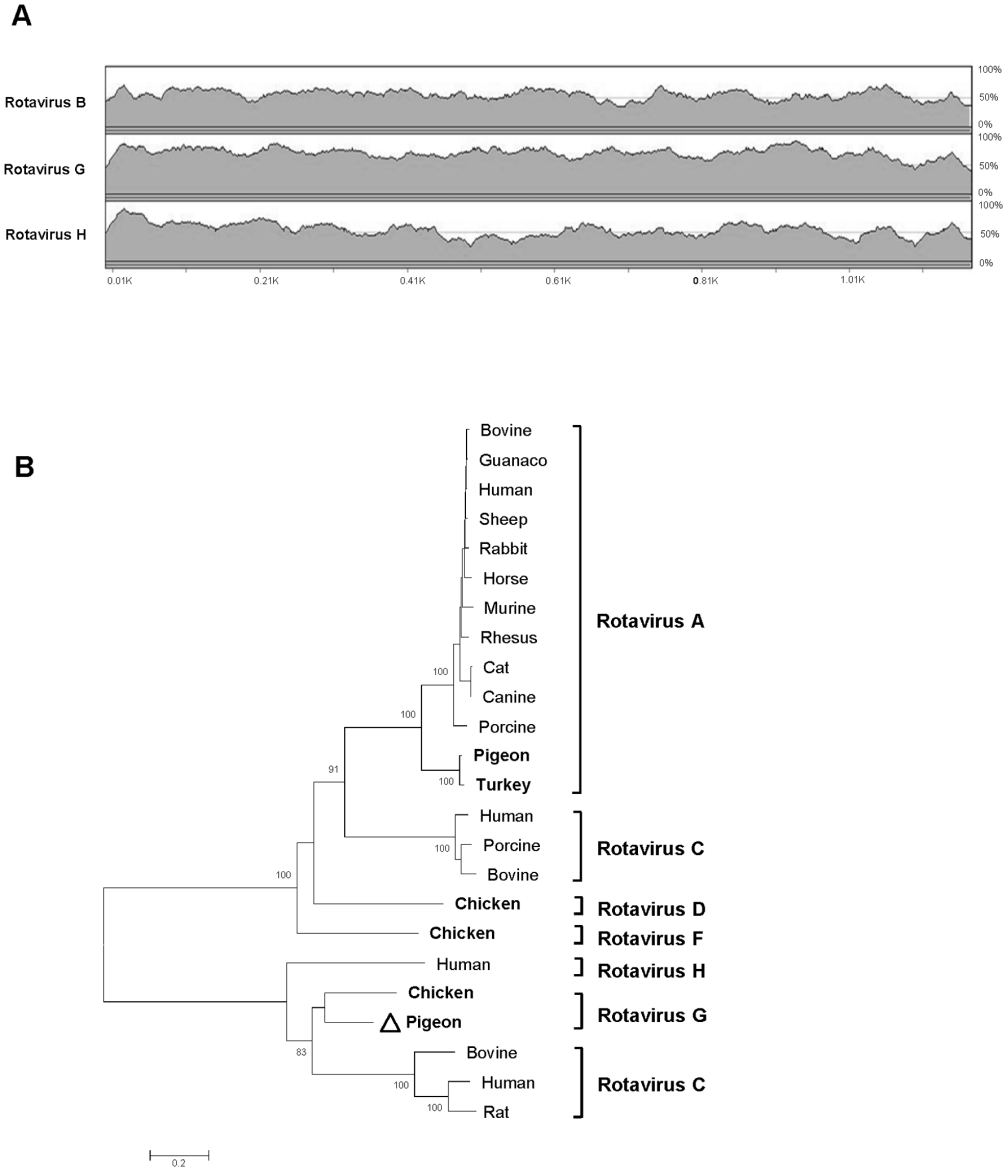
Pigeon rotavirus VP6 and phylogeny. **A.** Pair-wise sliding window of % nucleotide similarity of pigeon rotavirus VP6 gene aligned with the related rotavirus species. **B.** Phylogenetic analyses of VP6 protein of pigeon rotavirus and representatives of all rotavirus species. ICTV approved and proposed Rotavirus species are shown with those containing avian rotaviruses labeled in bold font. GenBank accession numbers used are included in Table S5.

**Figure 4:**
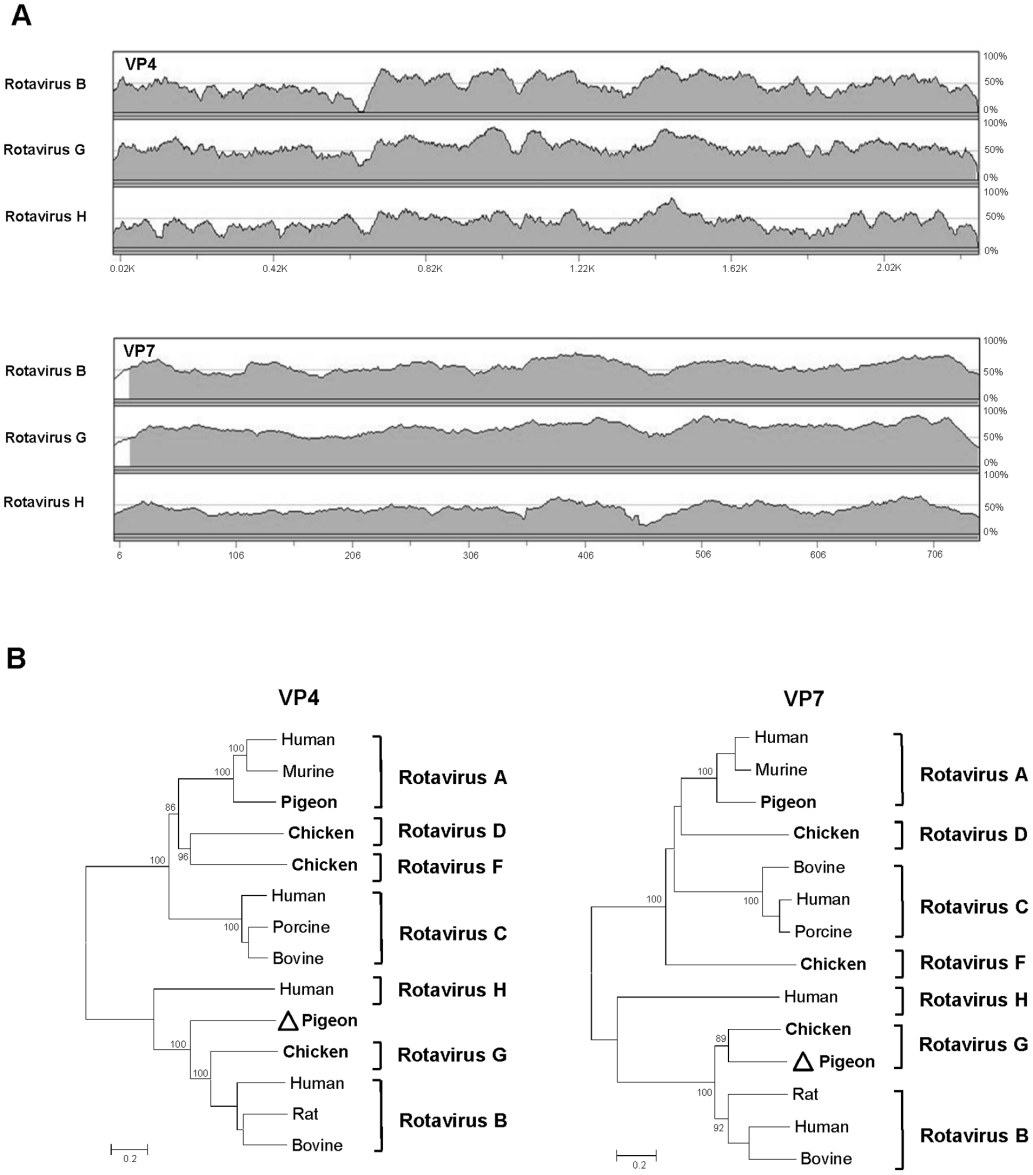
Pigeon rotavirus VP4, VP7 and phylogeny. **A.** Pair-wise sliding window of % nucleotide similarity of pigeon rotavirus VP4 and VP7 genes aligned with the related rotavirus species. **B.** Phylogenetic analyses of VP4 and VP7 proteins of pigeon rotavirus and representatives of all rotavirus species. ICTV approved and proposed Rotavirus species are shown with those containing avian rotaviruses labeled in bold font. GenBank accession numbers used are included in Table S5.

The six structural proteins (VP1–VP7) showed best hits by BLASTx to group G rotavirus, first described in chicken feces from Northern Ireland [81]. While VP1–VP3 shared 84%-94% aa-identities with group G rotavirus, other structural proteins showed lower aa-identities, ranging from 35% to 67% (Table 2). Classification of rotavirus is based mainly on the inner capsid protein VP6 and according to ICTV definition the members of the same rotavirus species should share >60% nt-identity in VP6 region [82]. The sequence (1176-nt) of the VP6-encoding genome segment shared 68% nt-identity to group G rotavirus (Figure 3A). Direct observation of VP6 alignment between HK18 and its closest relatives revealed numerous mismatches in the group-specific antigenic regions (Figure S3), indicating that HK18 represents a candidate prototype for a novel subgroup within group G. Two structural proteins VP4 (the protease-cleaved polyprotein) and VP7 (the glycoprotein) on the outermost surface of rotavirus are targets for neutralizing antibodies and have also been used to classify rotaviruses into their P and G genotypes, respectively [83]. The nucleotide sequence alignments of HK18 VP4 and VP7 showed low identity to other rotaviruses (Figure 4A). In addition, HK18 and group G rotavirus shared only 35% for VP4 and 51% for VP7 at the amino acid level, suggesting that HK18 should be considered as novel P and G genotypes, tentatively proposed as G2P [2] within group G rotavirus. The VP1 protein of HK18 contained three conserved RNA-dependent RNA polymerase motifs ^558^AEKII**LYTDVSQWD**A**S** ^573^, ^638^ **LKI**R**Y**LG**VASGEK**T**TK**IG**NS**F**AN**V**ALI** ^664^ and ^683^M**RVDGDDN**VVT^693^ [84]. Similar to group G rotavirus, the VP3 protein of HK18 did not possess the NTP-binding sequence motif present in group C rotavirus [85]. However, an alignment of VP3 sequences from different rotavirus groups showed that the VP3 protein of HK18 shared a high degree of conservation in ALYXLSN [ALY**S**LSN] motif, which has unknown function [86]. In order to phylogenetically classify pigeon rotavirus HK18, its six structural proteins were aligned to other group rotavirus representatives. While the VP1–VP3, VP6 and VP7 of HK18 were clustered with those of group G rotavirus (Figure 3B and Figure S4), its VP4 was located on a branch diverging closer to the root (Figure 4B).

**Table 2:** Amino acid sequence identities (%) of eleven proteins of a novel pigeon rotavirus to representatives of other rotavirus species (A-H) belonging to the genus *Rotaviru*s.

Pigeon	A	B	C	D	F	G	H
**Structural proteins**							
**VP1**	20	59	20	19	22	**91**	58
**VP2**	12	53	11	11	11	**94**	45
**VP3**	9	45	8	10	10	**84**	25
**VP4**	10	32	11	9	11	**35**	20
**VP6**	12	45	14	11	14	**67**	41
**VP7**	12	42	10	9	10	**51**	17
**Non-structural proteins**							
**NSP1** *	4	23	6	3	4	**19**	13
**NSP2**	10	60	12	12	11	**92**	46
**NSP3**	10	23	8	7	7	**64**	15
**NSP4**	14	16	10	6	9	**68**	6
**NSP5**	13	34	11	11	8	**50**	21

GenBank numbers of these viruses are available in Table S5.

* A major peptide NSP1–2 used for identity calculation.

The pair-wise amino acid sequence analysis demonstrated that nonstructural proteins NSP2–NSP5 shared top identities to group G rotavirus rather than other groups, ranging from 50% to 92% at the amino acid level (Table 2). However, NSP1 had a very low aa-identity, less than 31% to group G rotavirus. Like group G rotavirus, the NSP1 gene of HK18 had two ORFs encoding two minor and major peptides labeled as NSP1–1 (104-aa) and NSP1–2 (310-aa), respectively. While NSP1–1 had the best identity of 30% to group G rotavirus, NSP1–2 shared the closet match to group B rotavirus with 23% identity versus 19% to group G. The NSP1 zinc-binding domain could not be identified in HK18. The NSP2 was found to have a conserved sequence HGXGHXRXV and histidine triad (His-X-His-X-His-XX) located at the RNA binding domain [87]. Phylogenetic analysis of NSP2–NSP5 showed that the group G rotavirus was the closest relative to HK18 (Figure S4) in all fragments except for NSP1 where it appeared basal to both groups G and B. A similar observation was also made for avian group A rotaviruses where their NSP1 could not be grouped into taxonomic species [88].

## Discussion

Metagenomics has been used to analyze viral nucleic acids in feces collected from humans and a growing range of animals including primates, horse, bats, rodents, pigs, dogs, and turkeys [42], [58], [65], [69], [89]–[92]. We investigated viral sequences in 51 wild pigeon droppings followed by viral sequence similarity searches revealing sequences closely or distantly related to viral genomes in GenBank. The eukaryotic viruses detected included insect and plant viruses likely reflecting the pigeon diet as well as viruses already known to infect pigeons such as pigeon circovirus and adenoviruses. Also detected and characterized were novel genomes in viral families known to infect vertebrates.

Circovirus-like inclusion bodies were initially identified in the bursa of pigeons [93], [94] and the molecular epidemiology of pigeon circovirus worldwide has confirmed its importance as a pathogen associated with a wide range of illnesses in pigeon populations, including weight loss, respiratory distress and diarrhea [94]–[96]; possibly as a result of induced immunodeficiency aggravating the pathogenicity of co-infections such as those of adenoviruses [97]. Both pigeon circovirus and diverse adenovirus sequences were identified here.

Parvoviruses cause a variety of mild to severe symptoms in birds [98]–[101]. The *Parvoviridae* subfamily has recently undergone a large expansion in the number of know genera and species [58], [62], [102], [103]. Here a novel parvovirus was characterized in pigeon feces that was distantly related to chicken and turkey parvoviruses found in feces of farm animals with signs of enteric diseases [62], [98]. The pigeon parvovirus genome, the first from that host species, possessed an unusually long middle ORF that encoded a protein showing similarity to that encoded by an ORF of unknown function in the fowl adenovirus genome. This observation might reflect past lateral gene exchange between avian DNA viruses replicating in the nucleus. The pigeon, chicken and turkey parvoviruses cluster together phylogenetically, but have not yet been assigned to a *Parvovirinae* genus. We therefore propose a new genus provisionally called *Aviparvovirus* containing at least two species, pigeon parvovirus and the closely related turkey/chicken parvoviruses.

Two related picornaviruses (81% nucleotide similarity) from pigeons in Hong Kong and Hungary were also characterized and provisionally named *Mesivirus*-1 and -2 reflecting a wide geographic distribution of this viral clade. Based on the phylogenetic analysis of the most conserved region (P3) many of the bird picornaviruses clustered together (*Gallivirus, Orthoturdovirus, Paraturdivirus, Megrivirus,* and *Mesivirus*) in a supported clade that includes only the mammalian infecting *Kobuvirus* and *Salivirus* genera. It is therefore possible to speculate on the past existence of a strictly avian clade a member of which adapted to mammals later resulting in the *Salivirus/Kobuvirus* clade.

Rotaviruses were first described as a causative agent of gastroenteritis in humans in 1973 by using electron microscopy to examine biopsies of duodenal mucosa from children with acute non-bacterial gastroenteritis [104]. The first pigeon rotavirus was isolated from feces in 1983 and belongs to group A [105]. We describe here the complete coding regions of all eleven segments of a group G rotavirus, the first from pigeons, distinct from chicken rotavirus [85]. The addition of a second pigeon rotavirus genome to the viral database also shows that the diversity of known avian rotaviruses is likely to continue to expand with multiple rotavirus species infecting the same host species.

Animal species living in or around human habitation, including some bats and rodents species and pigeons, are recognized as the reservoirs of multiple zoonotic pathogens. Because of increasing contact between such peridomestic animals and humans a better understanding of these animals’ virome can inform future studies of their cross-species transmissions potential. Monitoring viral exchanges between pigeons and highly exposed humans using nucleic acids or serological assays for avian viruses will be facilitated by improved knowledge of their virome.

## Supporting Information

Figure S1 **Alignment of putative 2A1 sequences of Mesivirus-1 and Megrivirus.** B Phylogenetic trees of P1 and P2 regions of Mesivirus and other picornavirus genera in the family *Picornaviridae*.
(PDF)

Figure S2 **Alignment of N-ternimal amino acid (upper) and nucleotide (lower) sequences containing start and stop codons of two pigeon Mesiviruses.**
(PDF)

Figure S3 **Alignment of VP6 proteins of the novel pigeon rotavirus and other closely-related rotavirus species.**
(PDF)

Figure S4 **Phylogenetic trees of structural (shaded box) and non-structural proteins of the novel pigeon rotavirus and other rotavirus species (A-H).**
(PDF)

Table S1 **Pairwise amino acid sequence identities (%) between NS and VP regions of the novel pigeon parvovirus, turkey parvovirus and representatives of parvovirus genera.** GenBank numbers of these viruses are available in Table S3.
(PDF)

Table S2 **Coding potential/putative proteins of the genome of Mesivirus-1 and comparison of amino acid sequence identity (%) of its P1–3 and other closely-related picornaviruses in the** ***Picornaviridae*** **family.**
(PDF)

Table S3 **Representative members in the subfamily** ***Parvovirinae*** **for the phylogenetic trees in** **Figure 1** **and their GenBank numbers.** *Parvoviruses were used for pair-wise calculation in Table S1.
(PDF)

Table S4 **Representative members in the family** ***Picornaviridae*** **for the phylogenetic tree in** **Figure 2** **and their GenBank numbers.**
(PDF)

Table S5 **Representatives and their GenBank numbers in the genus** ***Rotavirus*** **for phylogenetic trees in** **Figures 2** **,** **4** **, S4.** * Rotaviruses were used for pair-wise calculation in Table 2.
(PDF)
