# Supplementary material for: The Viruses of Wild Pigeon Droppings
Source: PLoS One. 2013 Sep 4;8(9):e72787. doi: 10.1371/journal.pone.0072787 (PMC3762862; doi:10.1371/journal.pone.0072787)

A

```
Mesivirus-1  DTAQPRPFEKCEWPQQTESGIIFH---------------MPVRSHDPWTCQCVLCTCWRDDPITPEEQDW
Megrivirus   -SPTSETL..GDQVMLSVTMD.LDSIRELCTELMEGVDTLYG.ARAAKFAWM.QS.FEEWKKSDQDADEF

Mesivirus-1  FEDYGYYPFTVVDACRAG---------------FRENLESDDDTWEVQSLLDEIECTIMNQECD----NR
Megrivirus   .S.VSLVEPV.FQSPPPPSAREKAARLEEFFDYMYGCV..EEELHA.CDAVNAVIDSFYGSDG.FIGEI.

Mesivirus-1  ELFRELPEEYPEGPAFSGMWID--------------------TAGRWETSS-----LRGKEWYRLLCVG-
Megrivirus   DR.S...LS.....DHP.V.FRGEFYRCLYVTTQNRFFPQVAAYSPV..EHNLFALYEAMRQFE.M.IAD

Mesivirus-1  ------------------QFYDRPNERTIYDRQVRVYTPLAQER--------------------------
Megrivirus   PLSTSHHYQRLLFLLLQQ.WVIPEMKSM.REKLAFL..MV.HPERIFLDWNWELFHTQPFRYICNMVGVE

Mesivirus-1  ------------LSHQLQAQAERNLRIAGQAPVLARQMWIDTWTN-SVRYMAHGALGACVIGRMHEILNL
Megrivirus   TALTDDGFFSGDEEG.F..PTSCDQKFLNLVRAYRDA.IVNP.HTFE.C.ISFYD.TCRNDVPEWFKRAV

Mesivirus-1  LERVHDAFEAGRQADQLVDEGFSEESLPLCARGDWQEDDDFFSDGEEVDPPRFEG
Megrivirus   EDACF.TLC.RVAERVN.NNEVLI.RYRQLYDEIFS.EP.....-D.I.-AE.Q-
```

B

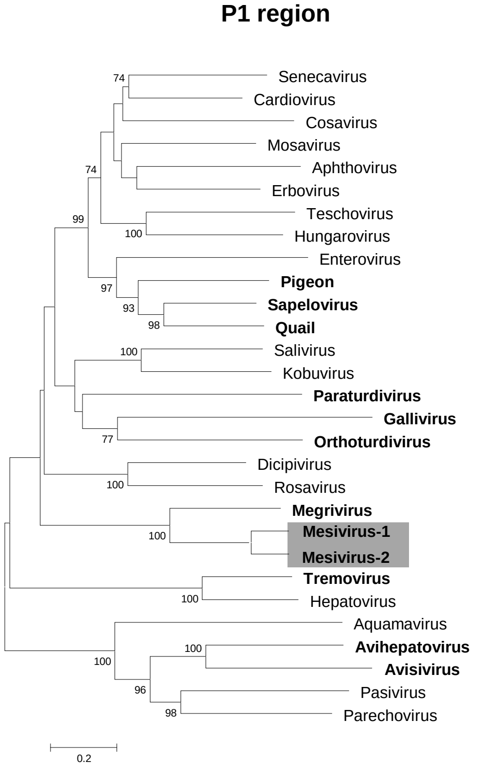

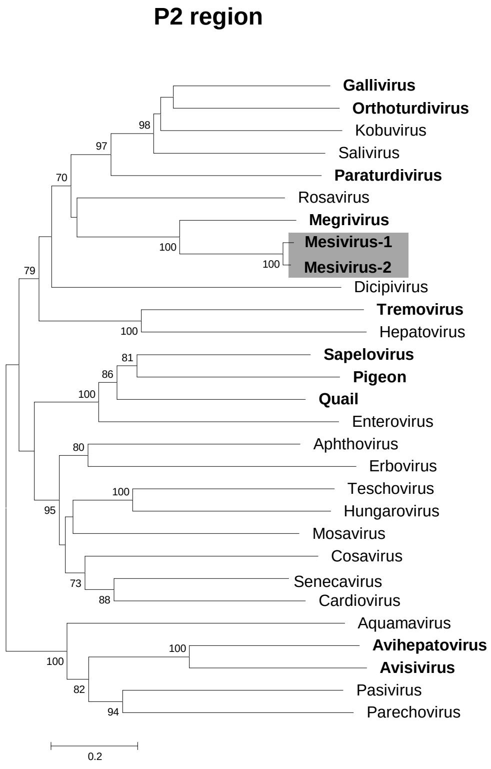

Supplement: Figure S1 — Alignment of putative 2A1 sequences of Mesivirus-1 and Megrivirus. B Phylogenetic trees of P1 and P2 regions of Mesivirus and other picornavirus genera in the family Picornaviridae. (PDF) [file pone.0072787.s001.pdf]
