# Supplementary material for: The Viruses of Wild Pigeon Droppings
Source: PLoS One. 2013 Sep 4;8(9):e72787. doi: 10.1371/journal.pone.0072787 (PMC3762862; doi:10.1371/journal.pone.0072787)
Supplement: Figure S2 — Alignment of N-ternimal amino acid (upper) and nucleotide (lower) sequences containing start and stop codons of two pigeon Mesiviruses. (PDF) [file pone.0072787.s002.pdf]

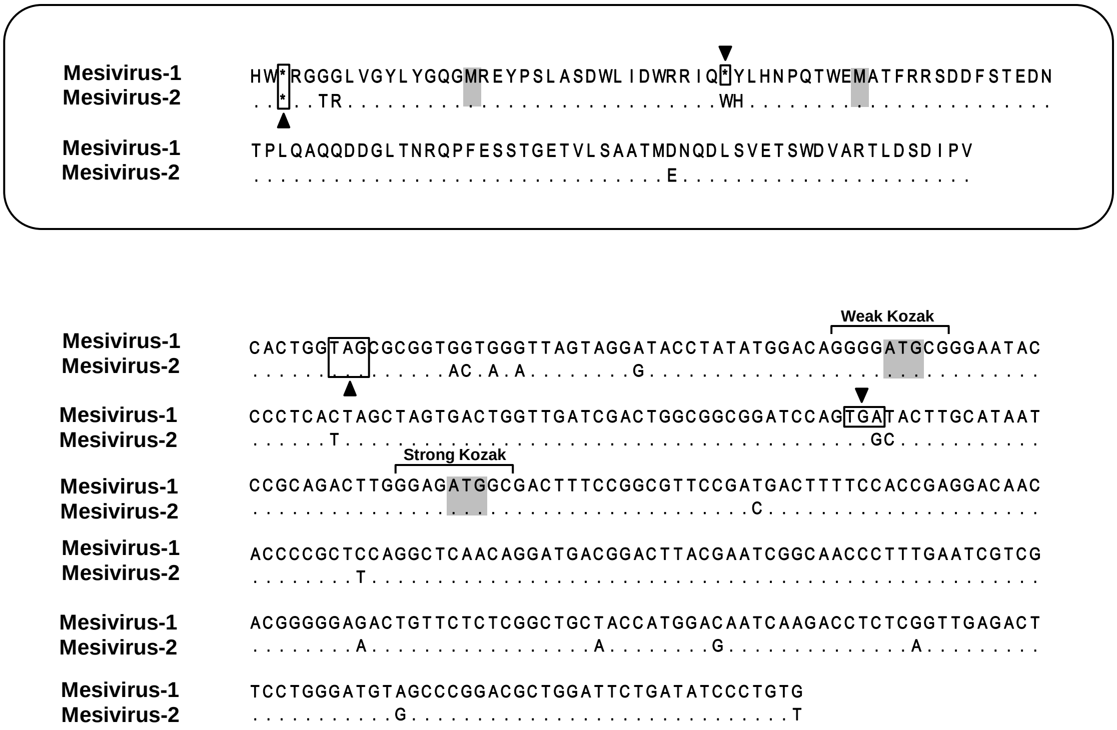
Mesivirus-1 HW*RGGGLVGYLYGQGMREYPSLASDWLIDWRRIQ*YLHNPQTWEMATFRRSDDFSTEDN
Mesivirus-2 ..*..TR............................WH.......................
Mesivirus-1 TPLQAQQDDGLTNRQPFESSTGETVLSAATMDNQDLSVETSWDVARTLDSDIPV
Mesivirus-2 ...............................E......................
Weak Kozak
Mesivirus-1 CACTGGTAGCGCGGTGGTGGGTTAGTAGGATACCTATATGGACAGGGGATGCGGGAATAC
Mesivirus-2 ...............AC.A.A........G..............................
Mesivirus-1 CCCTCACTAGCTAGTGACTGGTTGATCGACTGGCGGCGGATCCAGTGATACTTGCATAAT
Mesivirus-2 ......T........................................GC...........
Strong Kozak
Mesivirus-1 CCGCAGACTTGGGAGATGGCGACTTTCCGGCGTTCCGATGACTTTTCCACCGAGGACAAC
Mesivirus-2 ......................................C.....................
Mesivirus-1 ACCCCGCTCCAGGCTCAACAGGATGACGGACTTACGAATCGGCAACCCTTTGAATCGTCG
Mesivirus-2 ........T...................................................
Mesivirus-1 ACGGGGGAGACTGTTCTCTCGGCTGCTACCATGGACAATCAAGACCTCTCGGTTGAGACT
Mesivirus-2 ........A.................A........G..............A.........
Mesivirus-1 TCCTGGGATGTAGCCCGGACGCTGGATTCTGATATCCCTGTG
Mesivirus-2 ...........G.............................T
