# Supplementary material for: The Viruses of Wild Pigeon Droppings
Source: PLoS One. 2013 Sep 4;8(9):e72787. doi: 10.1371/journal.pone.0072787 (PMC3762862; doi:10.1371/journal.pone.0072787)
Supplement: Figure S3 — Alignment of VP6 proteins of the novel pigeon rotavirus and other closely-related rotavirus species. (PDF) [file pone.0072787.s003.pdf]

```
Pigeon rotavirus  MDLIETINAVVDLQKRVYKLSANTNLNSQGQQIINDYNALASRVSGKQYALLDQTAVLTPYLVDMGLVSI
Rotavirus B       ......V..C.K.....LG.AP.....TA..SVL..........N..T....N.I..Y...TINAPII.L
Rotavirus G       ......V.........I.E..S....S.K...VV..........N..T...H.........IIN.N.I..
Rotavirus H       ...........E..H..RN..P...IYQD..TT......I...CN..V.N.R..I.Q.S.FQ.H.PVIP.

Pigeon rotavirus  STRISTDDFDLMKDGMSSLFDAIAAAIRLECSRRVRAVEQRVLEPSVKMLLDDIKMKKQYSEITLANILA
Rotavirus B       AV......Y.D.RS.IE.IL.VL.....T.G..PT.VI.R.....N..Q.I..LRL.SLI.DLSI..LA.
Rotavirus G       ........YEA..E..NG.L.V......T........I.......V....IE..RLRA.V.K.AI..MS.
Rotavirus H       ..IL....HET.SA.IE....VL.....T.G..QN...VTKSI..E.LKAVL..GIRS.F..NPY..M.Q

Pigeon rotavirus  NDTAKLDNIIDKIENPLNPGIVDDVTYRPNFTQRGGGIRYTTGRWAGTRGAVTCVSGTDGEHVINIPLKT
Rotavirus B       V...MIQPEVIET....YAD.IEQ.VH...IGMT..N..A.L...S.NK.V...M..M.S..RFTVE...
Rotavirus G       Y.....EPQ.VN.....R.......LQ..................S.NK.I.........V.T.E.Q.R.
Rotavirus H       V....MEQQLEP.DD..ASQRINMLAAGGATNNT...YHALV..AT.KT.II.IIQ.RP.AVTF.LNM.V

Pigeon rotavirus  QMNGILSVMYIPAPGKISQSIG--RANGVPIALKCTDVSPDMTRNDITIEFVRSGVLIGTQRGTGAFQFK
Rotavirus B       RTC..INIV...TS.T.LIP----MPT.RNREGDLI...AE.MAD.FA.D.MDDDNI.Q.ET.V.VYS.P
Rotavirus G       TLT...NLL.V......V..R---..Q...VT.R.S......A.G..V...L.N.AVVDAEG.S.T...T
Rotavirus H       PCS.V..LTLL....V.QL.L.NQPP.NI.VHAE.V...TVF.EG..V.R.ELA.RVV.NANKL.TLN.P

Pigeon rotavirus  QANHIRIRVEPWNNAKNRN--PNPDFTNWNQNQANSQPTVSIMFEIMAAYAQVENDILASTDTKVQYYLD
Rotavirus B       MC.R..F.IN....Q.DDDNLGTVHMV..A.GT.PK..AI.F...TRRTFTEGDYQH.SRCAP.A..MM.
Rotavirus G       .CDT........SIV..L--.T...A.............L.....RQ..T.L...EMSADAP.......
Rotavirus H       LCDR.S.TI....AN.QN.--A.AN.N..PAGT.QR...I.LFVN.IN.SSLIDYETHSKYLAGAT.LMG

Pigeon rotavirus  THFTDDSFIRRP-NINWTVLDMLS--TQNDSIWAKKIAASVAAFSSKI
Rotavirus B       .Q.N.V..TN..-AVD.NIQSL.T--SNTQRV.CQ...MLI.S.AA..
Rotavirus G       .I..Q...V.H.-.VL.RTQ.LAN--A..NTA..R.VVT.....AA..
Rotavirus H       .T.SE....AS.P.VV..MSSL..GAPPQYIH.TR...CMI....C..
```
