# Supplementary figures and images for: The Viruses of Wild Pigeon Droppings
Source: PLoS One. 2013 Sep 4;8(9):e72787. doi: 10.1371/journal.pone.0072787 (PMC3762862; doi:10.1371/journal.pone.0072787)

**VP1**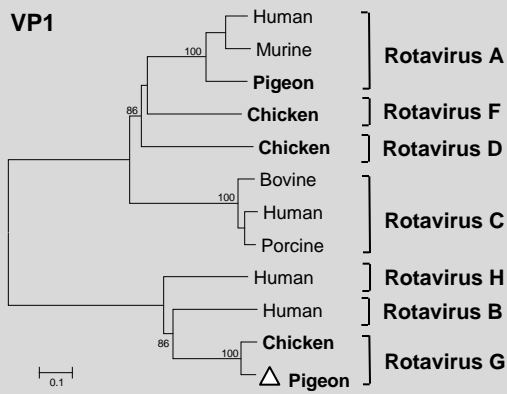**VP2**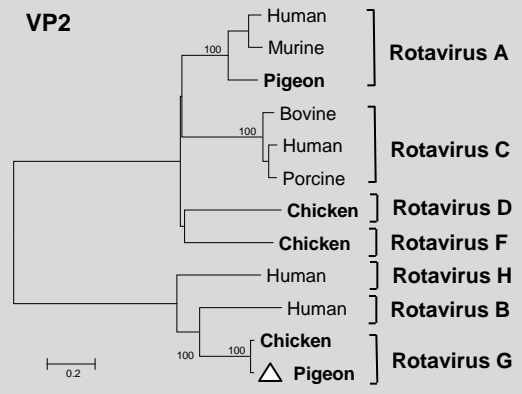**VP3**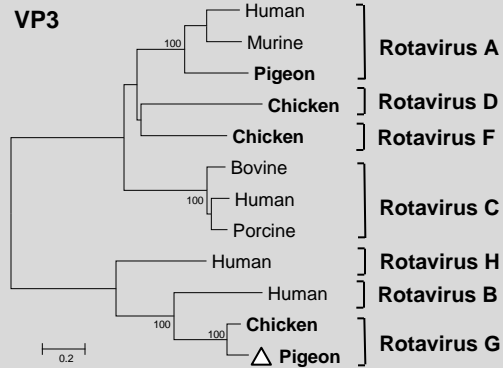**NSP1**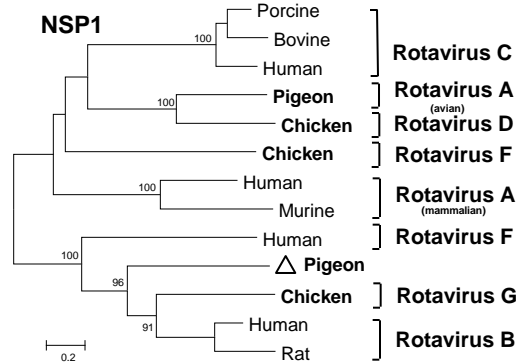**NSP2**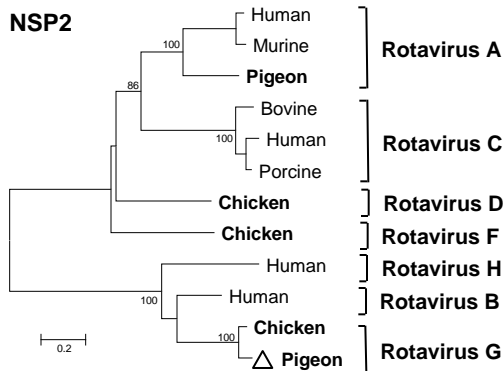**NSP3**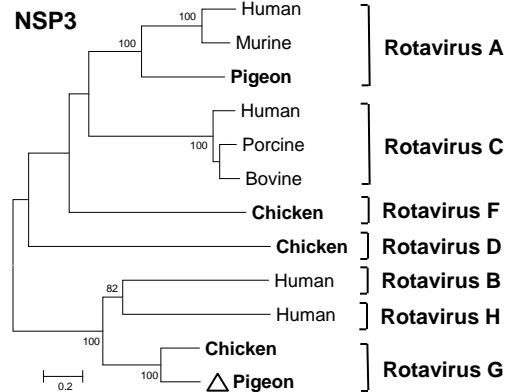**NSP4**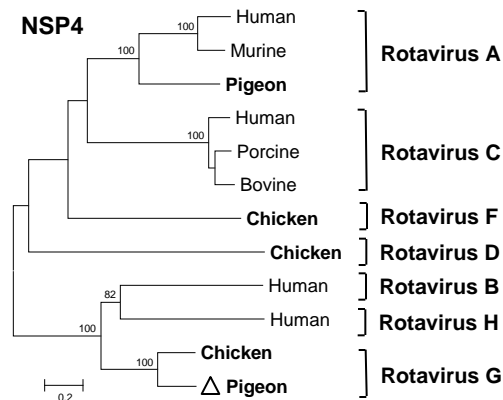**NSP5**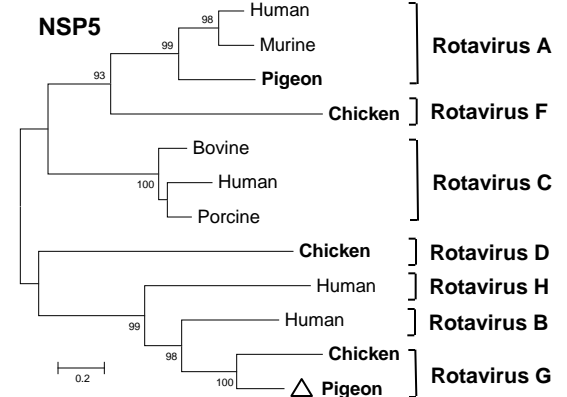

Supplement: Figure S4 — Phylogenetic trees of structural (shaded box) and non-structural proteins of the novel pigeon rotavirus and other rotavirus species (A-H). (PDF) [file pone.0072787.s004.pdf]
