# Supplementary material for: The Viruses of Wild Pigeon Droppings
Source: PLoS One. 2013 Sep 4;8(9):e72787. doi: 10.1371/journal.pone.0072787 (PMC3762862; doi:10.1371/journal.pone.0072787)
Supplement: Table S1 — Pairwise amino acid sequence identities (%) between NS and VP regions of the novel pigeon parvovirus, turkey parvovirus and representatives of parvovirus genera. GenBank numbers of these viruses are available in Table S3. (PDF) [file pone.0072787.s005.pdf]

	1	2	3	4	5	6	7
**NS**							
**1. Pigeon parvovirus**	ID						
**2. Turkey parvovirus**	**41**	ID					
**3. Bocavirus**	**20**	17	ID				
**4. Erythrovirus**	**16**	16	15	ID			
**5. Dependovirus**	**17**	17	15	20	ID		
**6. Amdovirus**	**14**	14	15	11	14	ID	
**7. Parvovirus**	**15**	14	16	13	18	18	ID
**VP**							
**1. Pigeon parvovirus**	ID						
**2. Turkey parvovirus**	**34**	ID					
**3. Bocavirus**	**18**	18	ID				
**4. Erythrovirus**	**16**	15	13	ID			
**5. Dependovirus**	**20**	22	24	19	ID		
**6. Amdovirus**	**10**	11	10	10	10	ID	
**7. Parvovirus**	**14**	13	19	10	17	28	ID
