# Supplementary material for: The Viruses of Wild Pigeon Droppings
Source: PLoS One. 2013 Sep 4;8(9):e72787. doi: 10.1371/journal.pone.0072787 (PMC3762862; doi:10.1371/journal.pone.0072787)
Supplement: Table S2 — Coding potential/putative proteins of the genome of Mesivirus-1 and comparison of amino acid sequence identity (%) of its P1–3 and other closely-related picornaviruses in the Picornaviridae family. (PDF) [file pone.0072787.s006.pdf]

Mesivirus-1			Megrivirus (HQ189775)	Salivirus (GQ179640)	Rosavirus (JF973686)	Kobuvirus (AB084788)
Putative protein	Position	Length (aa)				
**P1**			**43**	**16**	**15**	**15**
**VP0**	M_{1} - Q_{389}	389				
**VP3**	Y_{390} - Q_{557}	168				
**VP1**	G_{558} - Q_{807}	250				
**P2**			**34**	**12**	**19**	**15**
**2A1**	D_{808} - G_{1095}	288				
**2A2**	R_{1096} - Q_{1290}	195				
**2B**	A_{1291} - E_{1480}	190				
**2C**	A_{1481} - E_{1826}	346				
**P3**			**47**	**25**	**29**	**27**
**3A**	A_{1827} - E_{2008}	182				
**3B**	A_{2009} - E_{2036}	28				
**3C**	G_{2037} - Q_{2233}	197				
**3D**	G_{2234} - L_{2707}	474				
