# Supplementary material for: The Viruses of Wild Pigeon Droppings
Source: PLoS One. 2013 Sep 4;8(9):e72787. doi: 10.1371/journal.pone.0072787 (PMC3762862; doi:10.1371/journal.pone.0072787)
Supplement: Table S3 — Representative members in the subfamily Parvovirinae for the phylogenetic trees in Figure 1 and their GenBank numbers. *Parvoviruses were used for pair-wise calculation in Table S1. (PDF) [file pone.0072787.s007.pdf]

**Bocavirus**	Human* (HM132056)	Porcine (JN831651)	Canine (NC_004442)	Sea Lion (JN420365)	Gorilla (HM145750)	Bovine (DQ335247)
**Partetravirus**	Human* (AY622943)	Porcine (EU200677)	Bovine (EU200669)	Swine (AB076669)		
**Parvovirus**	Porcine* (FJ822038)	Rat (AF321230)	Canine (EF011664)	Mouse (NC_011618)	Rat (AF321230)	
**Dependovirus**	Human* (NC_001401)	Duck (NC_006147)	Goose (JF333590)			
**Erythrovirus**	Human* (AB550331)	Simian (U26342)				
**Amdovirus**	Mink* (JN040434)	Fox (JN202450)				
**Unclassified**	Turkey* (GU214706)	Chicken (EU304808)	Bovine (JF504697)	Bovine (AF406967)		
