# Supplementary material for: The Viruses of Wild Pigeon Droppings
Source: PLoS One. 2013 Sep 4;8(9):e72787. doi: 10.1371/journal.pone.0072787 (PMC3762862; doi:10.1371/journal.pone.0072787)
Supplement: Table S4 — Representative members in the family Picornaviridae for the phylogenetic tree in Figure 2 and their GenBank numbers. (PDF) [file pone.0072787.s008.pdf]

Gallivirus (JQ691613)	Orthoturdivirus (GU182406)	Kobuvirus (AB084788)	Salivirus (GQ179640)	Paraturdivirus (GU182408)
Megrivirus (HQ189775)	Dicipivirus (JN819202)	Rosavirus (JF973686)	Mosavirus (JF973687)	Teschovirus (AJ011380)
Hungarovirus (HM153767)	Erbovirus (X96871)	Aphthovirus (X00871)	Cosavirus (FJ438902)	Senecavirus (DQ641257)
Cardiovirus (M81861)	Enterovirus (X02316)	Sapelovirus (AY563023)	Quail (JN674502)	Pigeon (FR727144)
Pigeon (FR727145)	Tremovirus (AJ225173)	Hepatovirus (M14707)	Pasivirus (JQ316470)	Aquamavirus (EU142040)
Parechovirus (L02971)	Avihepatovirus (DQ219396)	Avisivirus (KC465954)		
