# Supplementary material for: The Viruses of Wild Pigeon Droppings
Source: PLoS One. 2013 Sep 4;8(9):e72787. doi: 10.1371/journal.pone.0072787 (PMC3762862; doi:10.1371/journal.pone.0072787)
Supplement: Table S5 — Representatives and their GenBank numbers in the genus Rotavirus for phylogenetic trees in Figures 2 , 4 , S4. * Rotaviruses were used for pair-wise calculation in Table 2. (PDF) [file pone.0072787.s009.pdf]

**Group A rotavirus**	Human*	Murine	Pigeon	Guanaco	Bovine	Sheep	Rabbit
Structural protein							
VP1	GQ414540	GQ479947	AB009629				
VP2	GQ414541	GQ479948	AB009630				
VP3	GQ414542	GQ479949	AB009631				
VP4	GQ414543	GQ479950	AB009632				
VP6	GQ414544	GQ479952	D16329	FJ347104	GU384194	EF554152	DQ205226
VP7	GQ414545	GQ479955	D82979				
Nonstructural protein							
NSP1	GQ414546	GQ479951	AB009633				
NSP2	GQ414547	GQ479954	AB009625				
NSP3	GQ414548	GQ479953	AB009626				
NSP4	GQ414549	GQ479956	AB009627				
NSP5	GQ414550	GQ479957	AB009628				
Group A rotavirus	Horse	Rhesus	Cat	Canine	Porcine	Turkey	
Structural protein							
VP6	JF712581	EF583013	EU708949	EU708916	AF317122	X98872	

**Group B rotavirus**	Human*	Rat	Bovine
Structural protein			
VP1	EU490415		
VP2	AY238390		
VP3	EU490418		
VP4	AY238388	P15155	GQ358710
VP6	AY238389	M84456	GQ358713
VP7	AY238385	P30889	AF531910
Nonstructural protein			
NSP1	AY238391		
NSP2	AY238393		
NSP3	AY238392		
NSP4	AY238384		
NSP5	AY238394		

**Group C rotavirus**	Human*	Porcine	Bovine	**Group D rotavirus**	Chicken*
Structural protein					
VP1	NC_007547	M74216	AB738412		NC_014511
VP2	AJ303139	M74217	AB738413		NC_014512
VP3	X96697	M74219	AB738414		NC_014514
VP4	NC_007572	M74218	AB738415		NC_014513
VP6	NC_007570	M94157	AB738416		NC_014516
VP7	NC_007571	M61101	AB738417		NC_014519
Nonstructural protein					
NSP1	AY238391	U01164	AY941785		NC_014515
NSP2	AJ132205	X65939	AB738408		NC_014518
NSP3	NC_007543	M69115	AB738409		NC_014517
NSP4	NC_007573	AF093202	AB738410		NC_014520
NSP5	M81488	X65938	AB738411		NC_014521

**Group F rotavirus**	Chicken*	**Group G rotavirus**	Chicken*	**Group H rotavirus**	Human*
Structural protein					
VP1	JN596591		JN596592		NC_007548
VP2	JQ919995		JQ920004		NC_007549
VP3	JQ919996		JQ920005		NC_007551
VP4	JQ919997		JQ920006		NC_007550
VP6	HQ403603		HQ403604		NC_007553
VP7	JQ919998		JQ920007		NC_007556
Nonstructural protein					
NSP1	JQ919999		JQ920009		NC_007552
NSP2	JQ920000		JQ920009		NC_007554
NSP3	JQ920001		JQ920010		NC_007555
NSP4	JQ920002		JQ920011		NC_007557
NSP5	JQ920003		JQ920012		NC_007558
